# A mobile one-lead ECG device incorporated in a symptom-driven remote arrhythmia monitoring program. The first 5,982 Hartwacht ECGs

**DOI:** 10.1007/s12471-018-1203-4

**Published:** 2018-12-06

**Authors:** J. L. Selder, L. Breukel, S. Blok, A. C. van Rossum, I. I. Tulevski, C. P. Allaart

**Affiliations:** 1 Amsterdam UMC, location VUMC, Amsterdam, Netherlands; 2 Onze Lieve Vrouwe Hospital, Amsterdam, Netherlands; 3 Cardiology Center Netherlands, Amsterdam, Netherlands

**Keywords:** eHealth, AliveCor, Kardia, Hartwacht, remote monitoring, ECG, arrhythmia

## Abstract

**Background:**

In recent years many mobile devices able to record health-related data in ambulatory patients have emerged. However, well-organised programs to incorporate these devices are sparse. Hartwacht Arrhythmia (HA) is such a program, focusing on remote arrhythmia detection using the AliveCor Kardia Mobile (KM) and its algorithm.

**Objectives:**

The aim of this study was to assess the benefit of the KM device and its algorithm in detecting cardiac arrhythmias in a real-world cohort of ambulatory patients.

**Methods:**

All KM ECGs recorded in the HA program between January 2017 and March 2018 were included. Classification by the KM algorithm was compared with that of the Hartwacht team led by a cardiologist. Statistical analyses were performed with respect to detection of sinus rhythm (SR), atrial fibrillation (AF) and other arrhythmias.

**Results:**

5,982 KM ECGs were received from 233 patients (mean age 58 years, 52% male). The KM algorithm categorised 59% as SR, 22% as possible AF, 17% as unclassified and 2% as unreadable. According to the Hartwacht team, 498 (8%) ECGs were uninterpretable. Negative predictive value for detection of AF was 98%. However, positive predictive value as well as detection of other arrhythmias was poor. In 81% of the unclassified ECGs, the Hartwacht team was able to provide a diagnosis.

**Conclusions:**

This study reports on the first symptom-driven remote arrhythmia monitoring program in the Netherlands. Less than 10% of the ECGs were uninterpretable. However, the current performance of the KM algorithm makes the device inadequate as a stand-alone application, supporting the need for manual ECG analysis in HA and similar programs.

## What’s new

- The AliveCor Kardia Mobile provides a patient-initiated 30-second one-lead ECG of diagnostic quality in ambulatory arrhythmia patients.
- For incorporation of a one-lead mobile ECG device in a regular healthcare setting, a dedicated arrhythmia program is of additional value as long as algorithms are not reliable enough.
- The future of remote monitoring for arrhythmias depends on custom-tailored algorithms. Refinement of detection of regular sinus rhythm in a way that it obviates the need for manual assessment of this category will reduce workload significantly

## Background

The healthcare system is a dynamic environment which evolves due to public demand and technological advancement. Currently, within the Netherlands, the system is changing to a state where patients are more responsible for and more in control of their own health, partly by the introduction of mobile health. Many devices and applications have been developed to provide patients with an opportunity to record specific health-related data outside the hospital, and heart rhythm has been an important area of interest in this. These devices are mostly targeted towards the general public and only few have been integrated in the healthcare system through a well-organised program with due regard for practicality, safety and privacy. An example of a well-incorporated device is the AliveCor Kardia Mobile (KM), a handheld ECG device, which is being used by the Hartwacht Arrhythmia (HA) program.

**Fig. 1:**
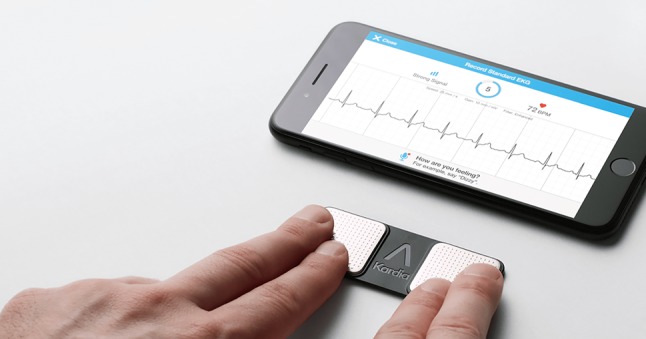
AliveCor Kardia Mobile device. (Downloaded with permission from www.Alivecor.com)

KM is a small ECG device, manufactured by AliveCor, which works by placing one finger of each hand on the electrodes (Fig. 1). A one-lead 30-second ECG (lead I) is recorded and transferred to a connected smartphone through a wireless communication protocol using ultrasonic audio. The ECG is simultaneously detected and locally analysed on the smartphone by the KM algorithm, which will classify it as normal sinus rhythm, possible AF, unclassified or unreadable. Both the ECG and its classification can then be sent on or emailed for further assessment.

The KM device and its algorithm have been assessed and validated in several studies. Sensitivity and specificity varied between 55–100% and 84–99% respectively, depending on the patient population and reference technique [1–6].

However, none of these studies have taken place in a real-life outpatient clinic setting. Hartwacht Arrhythmia, a remote monitoring program for heart rhythms in the Netherlands, initiated by Cardiologie Centra Nederland (CCN), provides an opportunity to evaluate the added value of the KM device incorporated in medical care. The HA program combines the KM with the expertise of a dedicated medical team led by a cardiologist for analysis of these ECGs, and thereby provides the opportunity to assess the accuracy of the KM algorithm in a real-life outpatient clinic setting as well as give insight as to the added benefit of this mobile device in the healthcare system. The aim of this study was to assess the benefit of the KM device in detecting cardiac arrhythmias in a real-world cohort of ambulatory patients, by analysing the results of the KM incorporated in the HA program focusing on the accuracy of the KM algorithm compared to KM interpretation of the Hartwacht team.

## Methods

### Study population

The study population consisted of all HA patients who submitted a KM ECG from the start of the program in January 2017 until March 2018. The Hartwacht program is available to patients of CCN, a private outpatient cardiology clinic. Typically, patients presenting with paroxysmal AF, palpitations of unknown origin or near-collapse were selected by the cardiologists of this clinic to participate in the Hartwacht program, although indications for inclusion in the program were left at the discretion of the physician.

### Hartwacht

After inclusion in the HA program, participants received the KM device at home, downloaded the Kardia smartphone application and were instructed on its use by the Hartwacht team. Whenever participants experienced palpitations or related complaints, they were encouraged to record an ECG with the KM device, after which the ECG and its classification by the algorithm were automatically transferred to the patient’s electronic patient record. There was no limit to the number of ECGs that could be recorded. ECGs were assessed by the Hartwacht team, consisting of a supervising cardiologist (0.05 FTE), a specialised cardiology nurse (1.0 FTE) and a doctor’s assistant (0.02 FTE), working on weekdays from 08.00 hrs to 17.00 hrs. Furthermore, a cardiologist who could directly access all Hartwacht ECGs was available 24/7 for emergency purposes. Patients received feedback from the Hartwacht team within one working day by phone or email.

### Data acquisition and analysis

Anonymised data were obtained from Hartwacht for analysis. Variables included in the analyses were patient characteristics, number of ECGs per patient per month and time of day that ECGs were received, classification of the ECG by the KM algorithm and results of assessment by the Hartwacht team. The KM algorithm classifies each ECG as one of four categories: (a) normal sinus rhythm, (b) possible AF, (c) unclassified or (d) unreadable. Assessment by the Hartwacht team resulted in classification as (a) sinus rhythm, with or without premature atrial contractions (PACs) and/or premature ventricular contractions PVCs, (b) atrial fibrillation, (c) other arrhythmias (including wide and narrow complex tachycardias and complete atrioventricular block) and (d) uninterpretable. Continuous variables were presented as mean ± standard deviation, categorical variables as frequencies. Sensitivity, specificity, positive predictive value (PPV), negative predictive value (NPV) and accuracy were calculated from the KM interpretation, with the Hartwacht team interpretation as reference standard. KM ECGs categorised as unclassified or unreadable were included in the sensitivity/specificity calculation when the Hartwacht team was able to provide a diagnosis. For example: An ECG interpreted by the KM algorithm as unclassified and subsequently by the Hartwacht team as atrial fibrillation deemed false negative for AF.

## Results

**Fig. 2:**
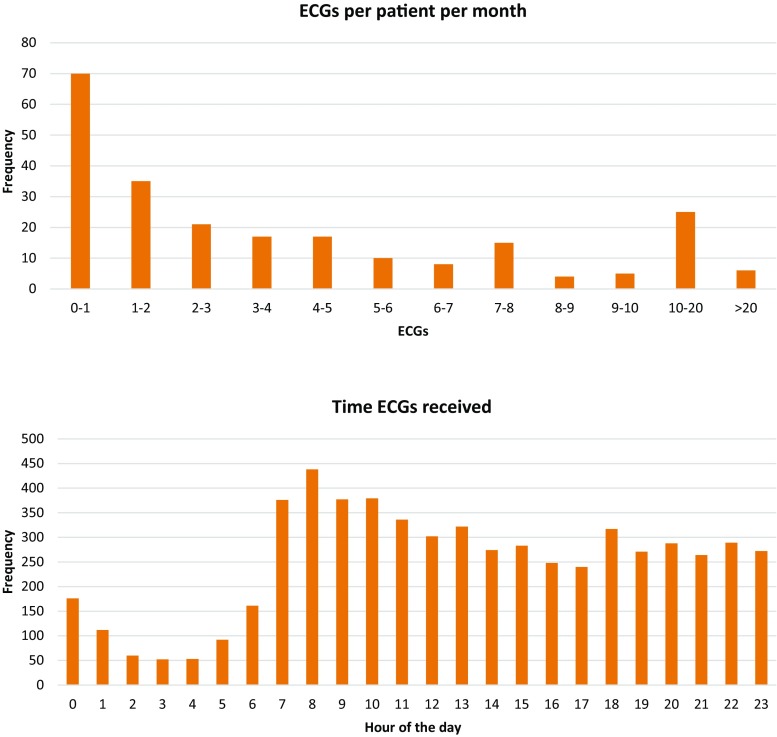
Number of KM ECGs received per patient per month and timing of received KM ECGs

**Fig. 3:**
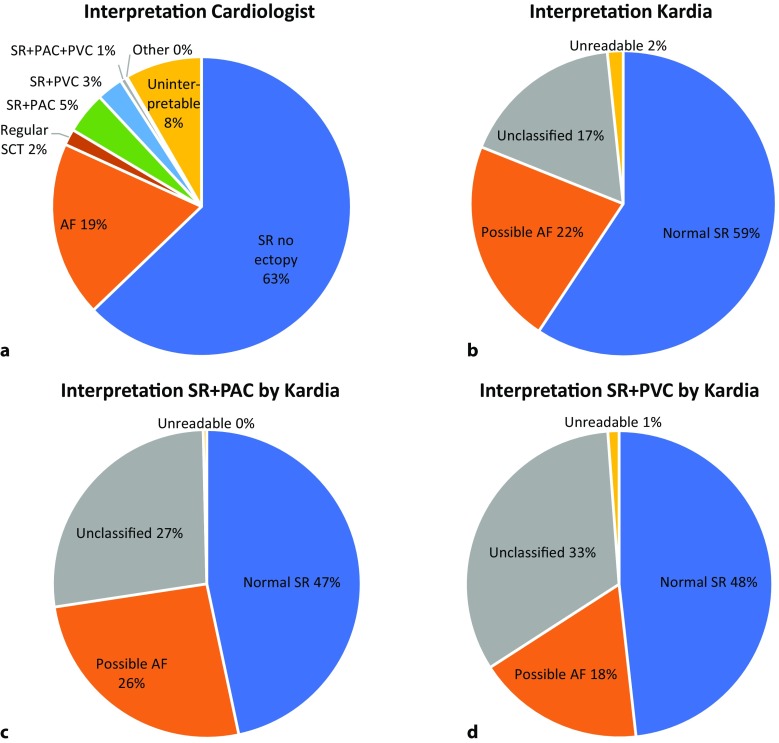
Interpretation of 5,982 KM ECGs (*SR* sinus rhythm, *AF* atrial fibrillation, *SCT* small complex tachycardia, *PAC* premature atrial complex, *PVC* premature ventricular complex)

**Fig. 4:**
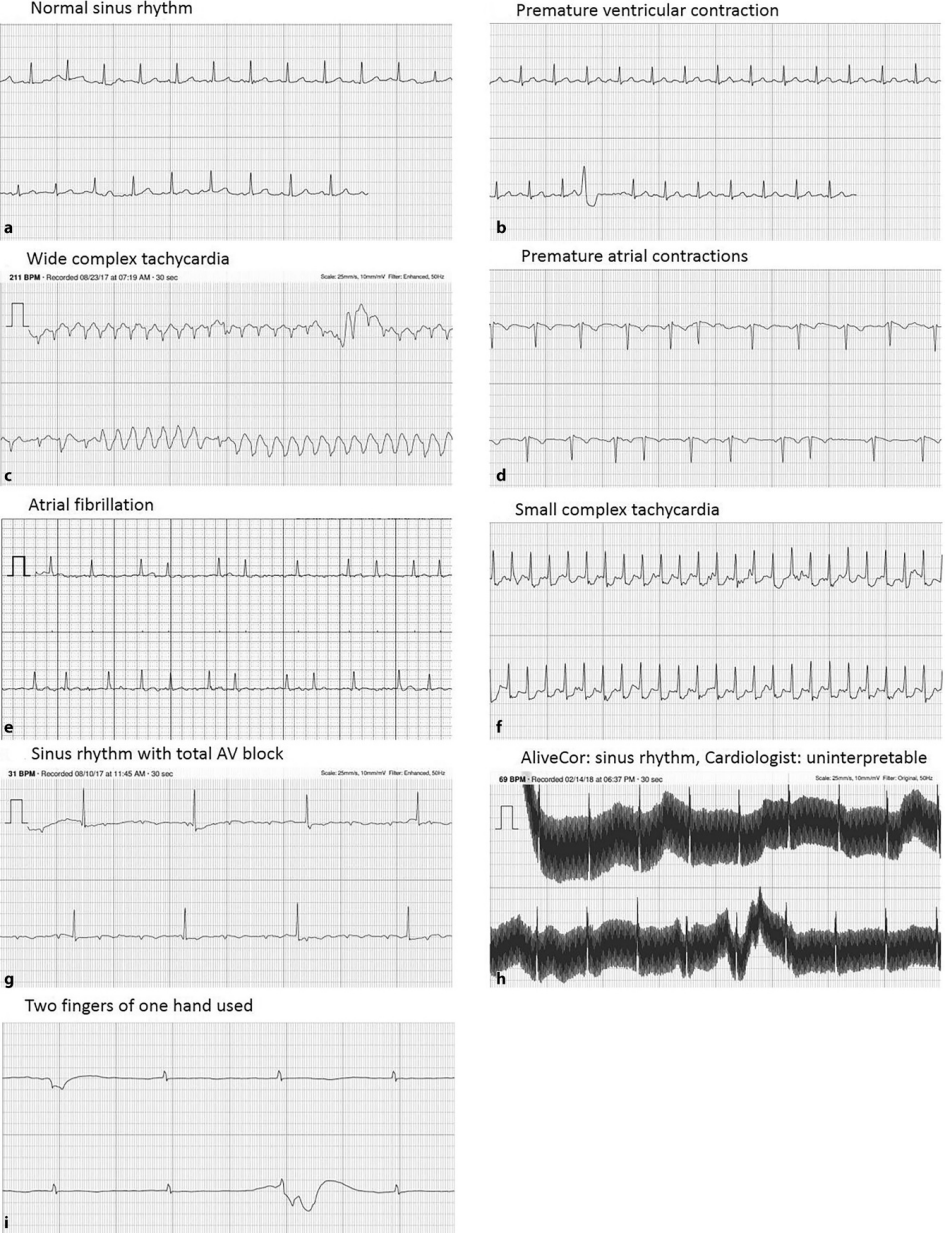
Various rhythms recorded with the KM device

A total of 233 participants in Hartwacht were included in the study. Patient characteristics are shown in Tab. 1. Seven patients (3%) exited the program, mostly because they never made ECGs. During the study period 5,982 KM ECGs were received, with a median of 28 ECGs per patient per year (Fig. 2a). Of these, the KM algorithm categorised 3,548 (59%) as normal sinus rhythm, 1,301 (22%) as possible atrial fibrillation, 1,033 (17%) as unclassified and 100 (2%) as unreadable (Fig. 3b). Analysis by the Hartwacht team resulted in 4,235 ECGs classified as sinus rhythm, of which 476 (8%) showed ectopy whereas 3,759 (63%) did not, 1,135 (19%) ECGs showed atrial fibrillation, 114 (2%) showed other arrhythmias and the remaining 498 were uninterpretable (8%) (Fig. 3a). Most ECGs were recorded between 07.00 and 11.00 hrs. A total of 3,023 ECGs (51%) were received outside office hours (17.00–08.00 hrs). Distribution of arrhythmias between classification groups in these ECGs did not differ from those received during office hours. Typical ECGs from every category are shown in Fig. 4.

**Table 1:** Patient characteristics

	*N* *=* 233
**demographics**	
age	58.4 (±14)
male	120 (52%)
**registered diagnoses**	
atrial fibrillation/flutter	127 (55%)
other supraventricular tachycardia	64 (28%)
ventricular tachycardia	24 (10%)
impulse and conduction disorder	10 (4%)
stable coronary artery disease	27 (12%)
acute coronary syndrome	3 (1%)
valvular heart disease	21 (9%)
chronic heart failure	5 (2%)
hypertension	62 (27%)
**anti-arrhythmic medication**	77 (33%)
amiodarone	2 (1%)
beta-blocker	17 (7%)
calcium channel blocker (verapamil, diltiazem)	23 (10%)
flecainide	14 (6%)
digoxin	14 (6%)
disopyramide	6 (3%)
sotalol	1 (0%)

Classification of the ECGs by the KM algorithm and diagnosis of the Hartwacht team differed significantly (Tab. 2). When the ECG was classified as sinus rhythm by the KM algorithm, the Hartwacht team agreed in 96% (90% without ectopy, 6% with ectopy); 4% were deemed uninterpretable and AF was diagnosed in <1%. When possible AF was detected by the KM algorithm, the Hartwacht assessment confirmed AF in 80% of cases. The remainder were diagnosed as sinus rhythm with or without ectopy (8% and 4%, respectively), other arrhythmias (1%) or uninterpretable (7%). From the ECGs that were classified by the KM algorithm as unclassifiable, the Hartwacht was able to provide a diagnosis in 81% of cases with 64% sinus rhythm (13% with ectopy), 8% atrial fibrillation, and 10% other diagnoses (including broad and small complex tachycardia and complete AV block). Even in the category unreadable, 29% of the ECGs could be interpreted by the Hartwacht team (Tab. 2).

**Table 2:** Interpretation of the ECGs by the KM algorithm (bold) and subsequent interpretation by the cardiologist (normal)

**normal sinus rhythm (KM)**	**3,548 (59%)**
atrial fibrillation	11 (<1%)
sinus rhythm	3,394 (96%)
– without ectopy	3,177 (90%)
– with PACs	126 (4%)
– with PVCs	82 (2%)
– with PACs and PVCs	9 (<1%)
other arrhythmias	2 (<1%)
– regular small complex tachycardia	2 (<1%)
uninterpretable	141 (4%)
**possible atrial fibrillation (KM)**	**1,301 (22%)**
atrial fibrillation	1,042 (80%)
sinus rhythm	162 (12%)
– without ectopy	49 (4%)
– with PACs	70 (5%)
– with PVCs	30 (2%)
– with PACs and PVCs	13 (1%)
other arrhythmias	10 (1%)
– regular small complex tachycardia	8 (1%)
– regular broad complex tachycardia	1 (<1%)
– complete AV block	1 (<1%)
uninterpretable	87 (7%)
**unclassified (KM)**	**1,033 (17%)**
atrial fibrillation	78 (8%)
sinus rhythm	658 (64%)
– without ectopy	515 (50%)
– with PACs	73 (7%)
– with PVCs	56 (5%)
– with PACs and PVCs	14 (1%)
other arrhythmias	97 (9%)
– regular small complex tachycardia	90 (9%)
– regular broad complex tachycardia	1 (<1%)
– complete AV block	6 (1%)
uninterpretable	200 (19%)
**unreadable (KM)**	** 100 (2%)**
atrial fibrillation	4 (4%)
sinus rhythm	21 (21%)
– without ectopy	18 (18%)
– with PACs	1 (1%)
– with PVCs	2 (2%)
other arrhythmias	4 (4%)
– regular small complex tachycardia	4 (4%)
uninterpretable	71 (71%)

### Ectopy and other arrhythmias

Irregular rhythms other than AF pose a challenge to the algorithm, which is illustrated by the distribution of ECGs diagnosed as sinus rhythm with ectopy over the KM classes. KM ECGs diagnosed by the cardiologist as sinus rhythm with PACs (5%) were classified by the KM algorithm as sinus rhythm (47%), atrial fibrillation (26%) or unclassified (27%) (Fig. 3c). ECGs with sinus rhythm and PVCs were similarly interpreted (Fig. 3d). In addition to sinus rhythm and atrial fibrillation, a small portion of ECGs (2%) showed other arrhythmias. For details see Tab. 2. There were several remarkable ECGs, shown in Fig. 4.

### Sensitivity and specificity

**Fig. 5:**
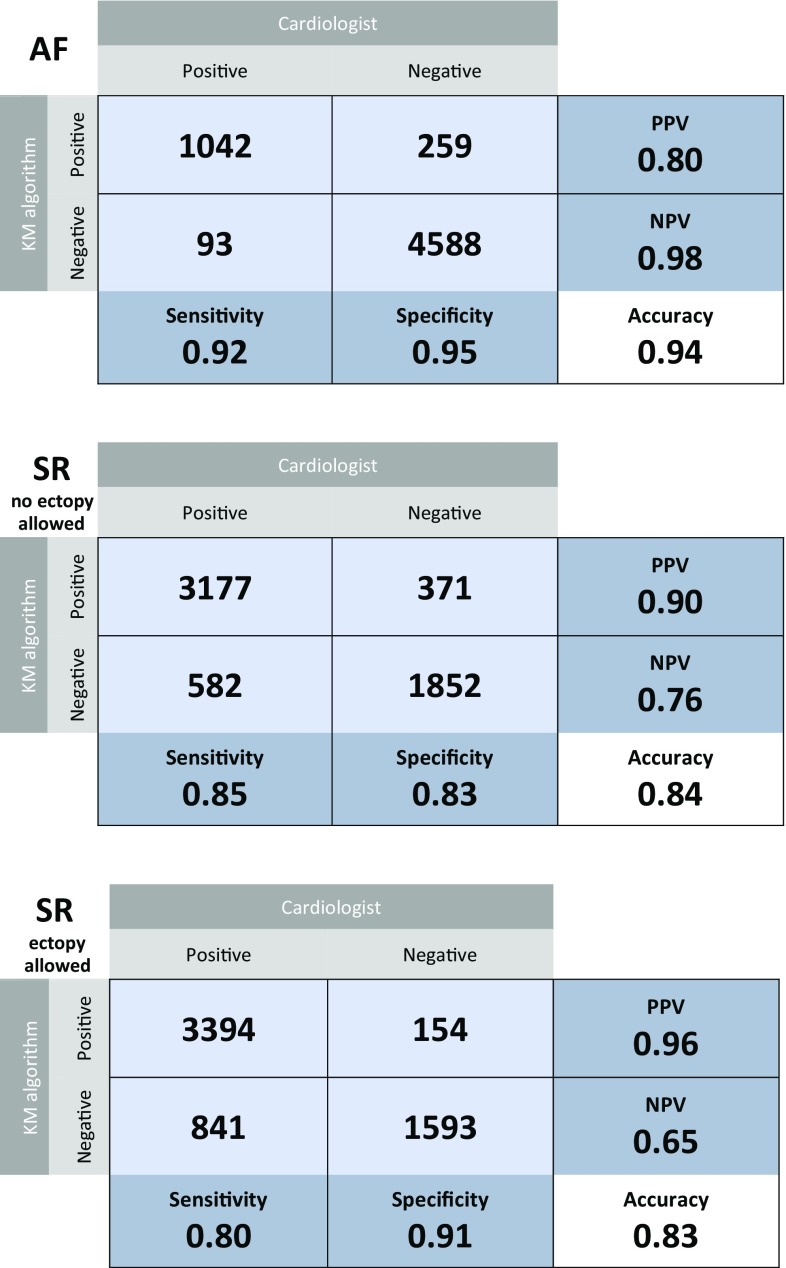
Two by two matrices of atrial fibrillation (*upper*) and sinus rhythm without (*middle*) or with (*lower*) ectopy

Using the assessment of the Hartwacht team as reference standard, the reliability of the different KM classifications in terms of sensitivity, specificity, PPV, NPV and accuracy, was determined (Fig. 5). For diagnosing AF these were 0.92, 0.95, 0.80, 0.98 and 0.94 respectively (upper table); for normal sinus rhythm (without any PACs or PVCs) 0.85, 0.83, 0.90, 0.76 and 0.84, (middle table); and for any form of sinus rhythm (with or without PACs or PVCs) 0.80, 0.91, 0.96, 0.65 and 0.83, respectively (lower table).

## Discussion

Hartwacht Arrhythmia is the first remote monitoring program in the Netherlands to use a device (KM) equipped with an algorithm to assess heart rhythm disorders. This study evaluated the ability of the algorithm to categorise 5,982 one-lead ECGs obtained with the KM device, and compared the outcome with the diagnosis provided by a dedicated arrhythmia team. The results show that erroneous classification by the algorithm in the category normal sinus rhythm was rare, assuming sinus rhythm with PACs and/or PVCs to be normal sinus rhythm (PPV 96%). On the other hand, ECGs classified as possible AF were diagnosed by the arrhythmia team as non-AF in 20% of cases, and in 81% of the unclassified ECGs the arrhythmia team was able to provide a diagnosis. These findings show that the KM/algorithm combination in its current form is inadequate for stand-alone clinical evaluation and support the need for additional assessment of the ECGs by an experienced ECG reader.

### Atrial fibrillation

The KM algorithm focusses on detection of AF. In previous studies validating the device, high sensitivity and specificity were obtained for detection of AF. Lau et al. compared the KM ECG to a 12-lead ECG in 109 cardiology patients, a third of whom were in AF, and found a sensitivity of 87% and specificity of 97% [1]. Using a similar study design, Haberman compared 381 athletes with healthy volunteers and cardiology clinic patients (sensitivity 94%, specificity 99%) [2]. On the other hand, screening of patients for AF in a geriatric ward resulted in a specificity of 98% but a disappointing sensitivity of 55%. The researchers partly attribute these findings to interference/artefacts resulting in poor *P*-wave recognition, which may be an issue in the elderly population.

In the present study, sensitivity and specificity for detection of AF were comparable to previously reported values (92% and 95%, respectively). PPV, however, was substantially lower (80%) and NPV was high (98%) compared to a previous study using the same algorithm [6]. Most likely, this is attributable to the relatively low prevalence of AF in our study cohort. Of note, comparison of outcomes between the present study and literature should be done with care since all ECGs categorised by the KM algorithm as unclassified or unreadable were often discarded in previous studies, whereas they were included in the present one. Although the majority of AF ECGs are classified correctly, 78 (7%) are erroneously allocated to the unclassified category and 11 AF (<1%) to the normal sinus rhythm category. In the present study, these ECGs were re-evaluated by the Hartwacht team, which significantly increased diagnostic yield with regard to AF and shows the added value of the HA program.

The KM device has also been studied as an AF screening instrument in asymptomatic patients aged 65 years or older [7–11], which resulted in 0.8–1.5% cases of newly diagnosed atrial fibrillation. The present study shows that when using the KM device for screening a general population for AF, two issues need to be considered. Firstly, prevalence of AF will be even lower compared to the present study and consequently PPV will be low with a high number of false positives resulting in a negative impact on cost-effectiveness. Secondly, a substantial number of AF ECGs will be not classified as possible AF emphasising the need for manual assessment of all ECGs.

### Ectopy and other non-AF arrhythmias

Sinus rhythm with ectopy (PACs and/or PVCs) provides a challenge to the algorithm. The algorithm is unable to categorise these arrhythmias properly, as is shown in Fig. 3c, d, where all three KM classes contain a substantial percentage of PACs and PVCs respectively. The importance of recognising these rhythms depends on the indication for participation in HA. Screening for AF would require these rhythms to be discarded whereas for analysis of palpitations of unknown origin, detection of SR with ectopy might provide a useful explanation. This might also apply for other rhythm disorders. Moreover, the algorithm classified 1,033 (17%) ECGs as unclassifiable. From 81% of those a diagnosis could be provided by the Hartwacht team, further supporting the need for manual evaluation of all ECGs in such a program.

### Limitations

Several limitations of this study need to be addressed. Firstly, this is a retrospective analysis of a patient population included in the HA program for various reasons and at the discretion of the physician. This may have introduced a substantial selection bias. Consequently, predictive values provided should be interpreted with caution as they vary with prevalence. Furthermore, in the present study the reference for analysis of the 30-second one-lead KM ECGs is the Hartwacht team. In a previous study, Bumgarner et al. reported a specificity of 97% for detection of AF when comparing the KM classification to interpretation by a cardiologist [6]. However, specificity dropped to 84% when comparing the KM classification to evaluation of a simultaneously obtained 12-lead ECG. As 12-lead ECGs were not available in the present study, specificity data might have been overestimated.

## Conclusion and future perspectives

The KM device provides a patient-initiated 30-second one-lead ECG of diagnostic quality in ambulatory arrhythmia patients. The present study shows the first remote monitoring arrhythmia program in the Netherlands. Less than 10% of the ECGs were uninterpretable. For detection of AF, the KM algorithm provides a high NPV, but PPV is relatively low, resulting in the need for manual assessment of all ECGs categorised as other than normal sinus rhythm. However, when the device is used for analysis of arrhythmias of unknown origin, all ECGs should be manually evaluated since non-AF arrhythmias (including ectopy) are poorly recognised by the algorithm and may be classified as normal sinus rhythm. Consequently, for incorporation in a regular healthcare setting a dedicated arrhythmia program is of additional value. The future of remote monitoring for arrhythmias will heavily depend on further enhancement of algorithms, leading to improvement of arrhythmia recognition. A practical first step might be the refinement of detection of regular sinus rhythm, which would obviate the need for manual assessment of this category of ECGs.
