# Correction to: A mobile one-lead ECG device incorporated in a symptom-driven remote arrhythmia monitoring program. The first 5,982 Hartwacht ECGs

**DOI:** 10.1007/s12471-019-1225-6

**Published:** 2019-01-23

**Authors:** J. L. Selder, L. Breukel, S. Blok, A. C. van Rossum, I. I. Tulevski, C. P. Allaart

**Affiliations:** 1 Amsterdam UMC, location VUMC, Amsterdam, The Netherlands; 2 Onze Lieve Vrouwe Hospital, Amsterdam, The Netherlands; 3 Cardiology Center Netherlands, Amsterdam, The Netherlands

**Correction to:**

**Neth Heart J 2018**

10.1007/s12471-018-1203-4

Unfortunately the original version of this article did not reflect that J.L. Selder and L. Breukel contributed equally to the study.

